# Immunocompromised patients with pulmonary tuberculosis; a susceptible group to intestinal parasites 

**Published:** 2018

**Authors:** Ali Taghipour, Taher Azimi, Ehsan Javanmard, Ali Pormohammad, Meysam Olfatifar, Ali Rostami, Payam Tabarsi, Mohammad Reza Sohrabi, Hamed Mirjalali, Ali Haghighi

**Affiliations:** 1 *Department of Parasitology and Mycology, School of Medicine, Shahid Beheshti University of Medical sciences, Tehran, Iran*; 2 *Department of Pathobiology, School of Public Health, Tehran University of Medical Sciences, Tehran, Iran*; 3 *Foodborne and Waterborne Diseases Research Center, Research Institute for Gastroenterology and Liver Diseases, Shahid Beheshti University of Medical Sciences, Tehran, Iran*; 4 *Department of Microbiology, School of Medicine, Shahid Beheshti University of Medical Sciences, Tehran, Iran*; 5 *Gastroenterology and Liver Diseases Research Center, Research Institute for Gastroenterology and Liver Diseases, Shahid Beheshti University of Medical Sciences, Tehran, Iran*; 6 *Infectious Diseases and Tropical Medicine Research Center, Health Research Institute, Babol University of Medical Sciences, Babol, Iran*; 7 *Department of Infectious Diseases, Mycobacteriology Research Center, National Research Institute of Tuberculosis and Lung Diseases (NRITLD), Masih Daneshvari Hospital, Shahid Beheshti University of Medical Sciences, Tehran, Iran*; 8 *Department of Community Medicine, School of Medicine, Shahid Beheshti University of Medical Sciences, Tehran, Iran*

**Keywords:** Tuberculosis, Immunodeficiency disorders, Intestinal parasites, Iran

## Abstract

**Aim:**

To investigate the presence of intestinal parasites in tuberculosis patients who suffered from immunodeficiency disorders.

**Background:**

Tuberculosis is an important infectious disease that is endemic in some regions of Iran. However, there is a coverage in the endemicity areas of this infection with intestinal parasites.

**Methods:**

Stool samples were collected from 50 immunocompromised tuberculosis patients. Direct smear using the normal saline (0.85% NaCl solution) and Lugol’s iodine staining were performed to detect trophozoite of parasites. Moreover, stool samples were concentrated using routine formalin-ether to detect protozoan cysts and helminth’s ova/larvae. Specific staining techniques including Trichrome, Modified Ziehl-Neelsen and chromotrope 2R were employed to detect amoeba, *Giardia *spp., coccidian parasites and microsporidia.

**Results:**

From 50 participants, 42 (84%) and 8 (16%) were male and female, respectively. The mean age + SD of patients was 47.88 + 10.88 years. Among the participated patients, HIV positive, cancer, organ transplant and receiving corticosteroids were seen in 13, 10, 15 and 12 subjects, respectively. The prevalence of Intestinal parasites was 34 %( 17/50). *Blastocystis *(18%; 9/50), and intestinal helminth (*Enterobius vermicularis*) (2%; 1/50) were the most prevalent and less prevalent parasites, respectively. Statistical significance difference was not seen between presence of intestinal parasites and type of immunodeficiency.

**Conclusion:**

Our findings showed the high prevalence of intestinal parasites with majority of *Blastocystis*. Indeed, this study suggested that due to complicated immune conditions of TB patients with immunodeficiency disorders, this group of patients are at higher risk of infection by intestinal parasites.

## Introduction

 During the years, pulmonary tuberculosis (TB) remains as one of the main reasons of death resulted from infectious diseases (1, 2). Based on the published reports of World Health Organization (WHO), the incidence rate of TB was 10 million with 1.3 million deaths among HIV-negative people and 300000 deaths among HIV-positive people. At the national level, the incidence of TB was 16 per 100000, In Iran (3, 4). In recent years, couple of studies investigated the co-infection of TB and intestinal parasitic infections (IPIs), including protozoans and helminths (5, 6). 

Based on published data, it seems that co-existence of these infectious agents has being emerged as a public health issue, particularly in developing countries (7, 8). Moreover, according to the worldwide reports of 2010, 438.9 million, 819 million and 467.6 million people were estimated to be infected with hookworms, *Ascaris lumbricoides*, and *Trichuris trichiura*, respectively (9). Presumably, concerning the vast distribution of intestinal parasites, socio-economic conditions are associated with the increase of the incidence of tuberculosis and IPIs in low-income populations (10-12).

On the other hand, presence of immunodeficiency disorders probably increase the complications in TB patients. The high prevalence of intestinal parasitic infections (IPIs) in immunocompromised TB patients (IC-TB) could be related with many factors such as lower CD4 T-cell count, poor hygiene, rural life, lack of nutritious foods and lack of refined drinking water (13-15). Importantly, intestinal helminths are able to strength Th2 immune response via induction of cytokines such as: IL-4, IL-5, and IL-13. Accordingly, change from Th1 toward Th2 during parasitic infection can pull down Th1 immune response to mycobacterium tuberculosis. Totally, the interaction between helminths and TB infections is unclear and controversial, thus so far to be completely understood (16, 17). 

Therefore, concerning the endemicity of TB and IPIs and emerging of immunodeficiency disorders, there is a little data in co-existence of TB, IPIs and immunodeficiency disorders. However, the current study aimed to investigate the prevalence of intestinal parasites among pulmonary tuberculosis patients who suffer from immunodeficiency disorders. 

## Methods

**Study population**

The current study received the ethic permission of the Ethics Committee of the Shahid Beheshti University of Medical Science (Number: IR.SBMU.MSP.REC.1395.323). Sample collection was conducted during February 2017 to May 2018 from Masih Daneshvari Hospital (the referral center for tuberculosis in Iran). A total of 50 IC-TB patients who were registered in the TB surveillance system and were undergoing anti-MTB treatment, participated in the current study. Prescription of anti-parasitic drugs during three-month prior to sample collection was considered as exclusion criterion. Stool samples were collected in a clean plastic containers and together with filled standard questionnaire (including demographic data, socio-economic features and risk factors related with IPIs) and a signed informed consent, were immediately transformed to parasitology laboratory. 

**Fecal sample collection and parasitological analysis**

In order to detect intestinal parasites, standard methods consisted of direct smear using the normal saline (0.85% NaCl solution) and Lugol’s iodine staining were performed for detection of protozoal trophozoite and cyst, respectively. Moreover, stool samples were concentrated using routine formalin-ether in order to detect protozoan cysts and helminth’s ova/larvae. Specific staining including Trichrome was employed to identify intestinal protozoans such as *Giardia lamblia and Entamoeba histolytica*. Modified Ziehl-Neelsen and chromotrope 2R staining were used for detection of *Cryptosporidium* spp. oocysts and microsporidia spores, respectively. All slides were examined under light microscopy (Zeiss, Germany) with 10×, 40× and 100× magnifications.

**Statistical analysis**

Data analysis was performed using STATA software version 14.2. The frequency and percentages of intestinal parasites and other descriptive were calculated by binomial distribution. The Chi square and Fisher's exact tests were used to compare prevalence of parasites among groups regarding socioeconomic risk factors.* P* values <0.05 were considered statistically significant. 

**Table 1:** Demographic characteristics and Risk Factors of IC-TBP, according to the IPIs

Variable	No tested (IC-TBP) (%)	Number of infected (%)	*P* value
Sex			0.08
Male	42 (82)	12 (70.58)	
Female	8 (18)	5 (29.42)	
Age			0.80
≤50	24 (48)	8 (47.05)	
>51	26 (52)	9 (52.95)	
Residence			0.08
Urban	35 (70)	5 (29.41)	
Rural	15 (30)	12 (70.59)	
Education			0.80
Primary school	14 (28)	9 (52.94)	
High school and above	36(72)	8 (47.06)	
Occupation			0.22
Employed	25 (50)	11 (64.70)	
Unemployed	25 (50)	6 (35.30)	
Consumption fruit and vegetable in rainy weeks			0.02^*^
Yes	14 (28)	7 (41.17)	
No	36 (72)	10 (58.83)	
Hand washing before eating			0.22
Yes	32 (64)	11 (64.70)	
No	18 (36)	6 (35.30)	

**Table 2:** Prevalence of the IPIs among immunocompromised patients who suffered from TB

Parasites	HIV/n=13 (N %)	Cancer/n=10 (N %)	Transplantation/n=15 (N %)	Consumption of corticosteroids/n=12 (N %)	Total/IC-TBP (N %)	*P* value
*Blastocystis *spp.	4 (30.76)	2 (20)	2 (13.33)	1 (8.33)	9 (18)	0.54
*Giardia lamblia*	1 (7.69)	1 (10)	0	0	2 (4)	-
microsporidia	0	1 (10)	0	0	1 (2)	-
*Cryptosporidium *spp.	1 (7.69)	1 (10)	1 (6.66)	0	3 (6)	-
*Entamoeba coli. *	0	0	1 (6.66)	0	1 (2)	-
*Endolimax nana*	1 (7.69)	1 (10)	2 (13.33)	0	4 (8)	0.77
*Trichomonas hominis*	0	0	1 (6.66)	0	1 (2)	-
*Chilomastix *	0	0	0	1 (8.33)	1 (2)	-
*Enterobious vermicularis*	1 (7.69)	0	0	0	1 (2)	-
Number of infected by protozoa	6 (46.15)	4 (40)	5 (33.33)	2 (16.66)	17 (34)	0.56
Number of infected by helminths	1 (7.69)	0	0	0	1 (2)	-

## Results

A total of 50 IC-TB patients including 13 HIV positive subjects, 10 cancer patients (receiving chemotherapies), 15 organ transplant recipients and 12 immunocompromised patients receiving corticosteroids participated in the current study. From 50 participants 42 (84%) and 8 (16%) were male and female, respectively with mean age (±SD) of 47.88±10.88 years. Demographic data and socioeconomic factors are summarized in table1 (Table 1). 

In addition, statistical analysis showed that there was a statistical significant correlation between consumption of fruits and vegetables in rainy seasons and presence of IPIs (*P *value = 0.02), while there was no statistical significant association between presence of intestinal parasites and other risk factors (Table 2). 

The results of parasitological examination showed the prevalence 34 %( 17/50) of IPIs among the patients. Accordingly, *Blastocystis *was the most prevalent IPIs (18%; 9/50) in all groups of IC-TB patients, while only one patient in HIV-TB group (2%; 1/50) was infected with intestinal helminths (*Enterobius vermicularis*). Moreover, the highest prevalence of IPIs was seen among those TB patients who were positive for HIV, while the less prevalence of IPIs was observed among TB patients who received immunosuppressive drugs. However, statistical significance difference was not seen between presence of intestinal parasites and type of immunodeficiency (Table 2).

## Discussion

TB and IPIs have been considered as public health issues in low- and middle-income countries. The results of present study showed that the total prevalence of IPIs among IC-TB patients was 34 %. This prevalence rate is approximately in accordance with previous comprehensive studies in Iran that showed the overall prevalence of IPIs among immunocompromised patients 11.7% (18), hemodialysis patients 30% (19) and patients with gastrointestinal disorders 32% (20). Moreover, the prevalence of IPIs in present study among IC-TB patients was similar to the results reported in Ethiopia as 40% (21). Interestingly, the prevalence of IPIs in the current study was higher than the study in China conducted by Li *et al. *who reported the prevalence of 7.3% (22) and 14.9% (10) in TB patients without HIV. This finding indicates that presence of immunodeficiency in TB patients can make them more susceptible to IPIs in comparison with TB patients without immunodeficiency and also healthy subjects. Furthermore, the results of other studies in Iran on the prevalence of IPIs in hemodialysis individuals (19, 23) and immunocompromised patients (18, 24, 25) support our findings. However, it was suggested that patients with immunodeficiency disorders are at higher risk of IPIs in comparison with immunocompetent subjects that the reason of this fact is correlated with insufficiency in immune response to the parasites in this group of patients (26). 

In the current study, the highest prevalence of IPIs was seen among TB patients who infected with HIV. However, there are studies that reported the role of HIV in shifting of immune response from Th1 toward Th2. Notably, defense against helminth parasites is mainly mediated by Th2 immune responses and thus, imbalance of immune response resulted from HIV, helminth parasites and/or both of them, makes human subjects more susceptible to TB (27, 28). On the other hand, co-existence of TB, intestinal parasites and HIV can be resulted from socioeconomic conditions of an area. In another words, all of these infectious agents are more prevalent in the regions with low level of hygiene and income. 

Apart from HIV that affects the immune system via an infectious agent, the other mentioned immunodeficiency disorders in the current study happened due to consumption of corticosteroids or immunesuppressor agents (29). However, there are several reports that indicated the role of consumption of corticosteroids or immunesuppressor drugs in modulation of immune response to infectious agents (30, 31). Although in the current we did not have data about the fact that TB or intestinal parasites/immunodeficiency disorders were firstly raised in the studied patients, it seems that presence of each of them can provide suitable conditions for infection by the other agents. In other words, consumption of immunosuppressor drugs attenuates overall immune response and makes human subjects prone to secondary infection by TB or intestinal parasites, particularly opportunistic infections (32).

Furthermore, findings of the current study showed that consumption of raw vegetables and fruits, particularly in rainy seasons, increases the risk of IPIs in susceptible subjects especially TB patients who suffer from immunodeficiency disorders. The increased risk of transmission of intestinal parasites to human via eating raw vegetable and fruits were frequently reported. Bekele and colleagues showed that 54.4% of vegetable and fruit samples were contaminated with intestinal parasites (33). In Iran, Asadpour and colleagues reported that 36.8% of farm vegetables were contaminated with intestinal parasites (34). In another study, Javanmard and colleagues showed that 41.7% of farm vegetables were seen contaminated with intestinal parasites (35). The high prevalence of intestinal parasites in raw vegetable and fruits supports our finding that showed statistical correlation between consumption of raw vegetables and presence of intestinal parasites in a susceptible group such as TB patients with immunodeficiency disorders. 

However, it is proposed that co-existence of TB and acquired immune deficiencies provides a complicated immune conditions that make patients more prone to intestinal parasites. In addition, socio-economic conditions, especially in developing countries can enhance the risk of co-infection of TB and intestinal parasites. 

In the current study, intestinal parasites were observed amongst 34% of TB patients who suffer from immunodeficiency disorders. In addition, the highest prevalence of IPIs was seen in TB patients with HIV. However, TB patients due to immune response to *Mycobacterium tuberculosis, *experience complicated condition that make them more prone to particularly helminths parasites. This intricate issue would be worst if this group of patient suffers from an immunodeficiency disorder. Therefore, it seems that TB patients with immunodeficiency are probably more susceptible to gastrointestinal infections, particularly parasites. Thus, providing a suitable personal hygiene and also regular health checkup for intestinal parasites are recommended.
